# Preventive Role of Salsalate in Diabetes Is Associated With Reducing Intestinal Inflammation Through Improvement of Gut Dysbiosis in ZDF Rats

**DOI:** 10.3389/fphar.2020.00300

**Published:** 2020-03-19

**Authors:** Xinrong Zhang, Xiao Cui, Xiaofang Jin, Fei Han, Jingyu Wang, Xiaoyun Yang, Jie Xu, Chunyan Shan, Zhongai Gao, Xiaochen Li, Minxia Zuo, Juhong Yang, Baocheng Chang

**Affiliations:** ^1^NHC Key Laboratory of Hormones and Development, Tianjin Medical University, Tianjin Key Laboratory of Metabolic Diseases, Tianjin Medical University Chu Hsien-I Memorial Hospital and Tianjin Institute of Endocrinology, Tianjin, China; ^2^Tianjin Medical University General Hospital, Tianjin Medical University, Tianjin, China

**Keywords:** gut dysbiosis, inflammation, leaky gut syndrome, salsalate, type 2 diabetes

## Abstract

A safe and effective approach is needed to prevent and reduce the incidence of diabetes worldwide. The hypoglycemic efficacy of salicylic acid (salsalate, SAL), which has anti-inflammatory properties, has been empirically demonstrated in studies conducted at the Joslin Diabetes Center and elsewhere. Here, we investigated the potential role of SAL in preventing the onset of diabetes in Zucker diabetic fatty (ZDF) rats and attempted to elucidate its underlying mechanisms. ZDF and Zucker lean (ZL) rats were administered a high-fat diet with or without SAL intervention, and their relative rates of diabetes were compared. Our results showed that all rats in the placebo group developed diabetes, whereas only 10% of the SAL-treated rats presented with impaired glucose tolerance (IGT). None of the latter progressed to diabetes. Relative to the untreated rats, SAL lowered plasma glucagon and insulin while improving insulin sensitivity and β-cell function. SAL may protect against hyperglycemia by increasing the microbial diversity, ameliorating gut dysbiosis, restoring intestinal epithelial cell connections, inhibiting endotoxin influx into the blood, and attenuating inflammation. Together, these findings suggest that SAL may be a candidate prophylactic therapy against diabetes. The protective role of SAL may be attributed to its ability to reduce intestinal inflammation and improve gut dysbiosis.

## Introduction

According to International Diabetes Federation (IDF) data, as of 2017, there were 451 million people with diabetes worldwide. The number of diagnosed cases is expected to rise to 693 million by 2045 (Cho et al., 2018). It was also estimated that there are 374 million people with impaired glucose tolerance (IGT) who are at high risk of becoming diabetic (Cho et al., 2018). Type 2 diabetes (T2D) has become a major human health challenge and is a global health burden because of its high morbidity and mortality. Therefore, measures that can effectively prevent or delay the onset of T2D are urgently needed (Defronzo et al., 2013; Hemmingsen et al., 2017).

There is growing evidence that T2D is associated with low-grade inflammation. Gut dysbiosis and the influx of microbial factors have been linked to inflammation and impaired glucose metabolism (Cani et al., 2007; Henao-Mejia et al., 2012). Recent research has focused on elucidating the causal relationships among these three factors. Lipopolysaccharide (LPS)-containing microbiota and metabolic endotoxemia initiate obesity and insulin resistance (Fernandes et al., 2019; Pascale et al., 2019). Moreover, hyperglycemia significantly interferes with epithelial barrier function, which leads to an abnormal influx of immunostimulatory microbial products and the diffusion of intestinal pathogens (Thaiss et al., 2018). In “leaky gut syndrome”, dysbiosis may increase intestinal permeability, activate the innate immune system, modulate lipid and glucose metabolism, trigger low-grade inflammation, cause insulin resistance, and induce T2D (Sabatino et al., 2017). These findings have raised the hope of identifying anti-inflammatory agents that are valid therapeutic targets for T2D.

SAL is a nonacetylated salicylate. It is routinely administered as anti-inflammatory agent (Reymond and Farmer, 1998). Its anti-inflammatory mode of action consists of inhibiting prostaglandin synthesis by inactivating cyclooxygenases (Higgs et al., 1987). As early as 1876, Ebstein reported that SAL might have antidiabetic efficacy. Several recent studies confirmed the hypoglycemic effects of SAL in diabetic mice and human patients (Barzilay et al., 2014; Nie et al., 2017). The anti-inflammatory action of SAL is a principal mechanism of improving glucose homeostasis (Liang et al., 2015). By inhibiting β-cell dedifferentiation, SAL reversed hyperglycemia and restored islet function in diabetic rats (Han et al., 2019a). However, the exact mechanism by which SAL prevents T2D has not been clarified.

In the present study, we assessed the effects of SAL on blood glucose, intestinal inflammation, and gut flora composition in Zucker diabetic fatty (ZDF) rats. We also attempted to elucidate the biochemical mechanism of SAL and focused primarily on intestinal microbiota changes, metabolic endotoxemia, and gut microflora structure.

## Materials and Methods

### Experimental Animals

All animal experiments complied with the rules of the Experimental Animal Care and Use Center at Tianjin Medical University, China. The protocols were approved by the Experimental Animals Ethical Committee of Tianjin Medical University. ZDF rats (*n* = 26) aged 6 weeks served as the experimental group and age-matched Zucker lean (ZL) rats (*n* = 10) were used as the normal control group. Rats were housed at room temperature (20–25°C) and relative humidity (RH) = 50–70%. They were maintained under a 12-h light/12-h dark cycle. All animals had ad libitum access to water and were fed with Purina 5008 chow consisting of 23% (w/w) protein, 6.5% (w/w) fat, 58.5% (w/w) carbohydrates, 4% (w/w) fiber, and 8% (w/w) ash.

There are literatures that indicate that intervention at 8 weeks can prevent T2D development by improving inflammation (Teixeira-Lemos et al., 2011; Teixeira De Lemos et al., 2011). Based on literatures and our results, at age 9 weeks, ZDF rats were randomly assigned either to the untreated ZDF rats or the ZDF+S group. The rats in the ZDF+S group were administered SAL (50 mg kg^−1^ day^−1^ p.o.) purchased from Med Chem Express (Monmouth Junction, NJ, USA). The purity of the SAL powder was 99%. The untreated ZDF rats and ZL rats were administered saline orally. After 3 weeks, the status of glucose tolerance in various groups was checked on the basis of oral glucose tolerance test (OGTT) results. The diagnostic criteria are indicated below. The rats were euthanized at 6 weeks, 9 weeks, and 12 weeks. Fresh fecal samples were collected and frozen at −80°C for microbiota analysis.

### Oral Glucose Tolerance Test

Rats were fasted overnight (12 h) and subjected to glucose (2 g kg^−1^ BW) loading at 6 weeks, 9 weeks, and 12 weeks. Blood was then collected from their tail veins. Glucose levels were measured at 0 min, 30 min, 60 min, and 120 min after glucose administration and the following calculations were made:

$$\text{AUCg}=({\text{G}}_{0}+{\text{G}}_{120})/4+({\text{G}}_{30}+{\text{G}}_{60})/4({\text{G}}_{60}+{\text{G}}_{120})/2$$

The homeostatic model assessment index of β-cell secretion (HOMA-β) = 20 × FINS/(G_0_ − 3.5)

The homeostatic model assessment index of insulin resistance (HOMA-IR) = G_0_ × FINS/22.5.

where AUCg (mmol/L) is the area under the curve for glucose, G is the blood glucose level (mmol/L) (0 min [G_0_]; 30 min [G_30_]; 60 min [G_60_]; and 120 min [G_120_]), and FINS (mU/L) is the fasting plasma insulin.

### Diagnostic Criteria

The diagnostic criteria for normal glucose tolerance, IGT, and DM were based on the OGTT results. DM was diagnosed for rats with peak plasma glucose > 16.8 mM and 120-min plasma glucose > 11.2 mM. IGT was diagnosed for rats with either one of the mentioned conditions. Rats with neither DM nor IGT were considered normal (Kawano et al., 1992).

### Blood Biochemical Markers

Rats were fasted overnight and blood samples were drawn from their femoral arteries at 6 weeks, 9 weeks, and 12 weeks. Total cholesterol (TC), triglyceride (TG), low-density lipoprotein cholesterol (LDL-C), and high-density lipoprotein cholesterol (HDL-C) levels were determined with an automatic biochemical analyzer. Fasting plasma insulin and glucagon and serum LPS were determined with enzyme-linked immunosorbent assay (ELISA) kits (Hermes Criterion Biotechnology, Vancouver, BC, Canada).

### Tissue Collection and Processing

Ileum samples were separated at 6 weeks, 9 weeks, and 12 weeks, and the fecal matter in them was cleared with phosphate-buffered solution (PBS). The ileal tissue was rapidly separated into four parts. One was immersed in liquid nitrogen, one was fixed in 4% (v/v) paraformaldehyde, one was fixed in 2.5% (v/v) glutaraldehyde, and the last was used for supernatant extraction and analysis. The ileum supernatant was collected by centrifugation (8000 × *g*, 15 min, 4°C) and stored at −80°C. The LPS level in the ileum supernatant was determined with an ELISA kit (Hermes Criterion Biotechnology, Vancouver, BC, Canada).

### Gut Microbiota Analysis by 16S rRNA High-Throughput Sequencing

Genomic DNA was extracted from the rat feces as previously described (Kozich et al., 2013). Relative fecal bacterial abundance and ratios were determined by high-throughput 16S rDNA V3-V4 DNA sequencing. Amplicons of the 16S rRNA gene V4 region were sequenced on the Illumina MiSeq platform (BGI Genomics, Shenzhen, China).

According to the results of the operational taxonomic unit (OTU) evaluation, alpha and beta diversity analyses were performed for all samples to determine species richness, evenness, and differences in bacterial community structure. Alpha diversity was assessed using Chao, observed species, Shannon, Ace, and Simpson indices. Greater values for the first four indices and smaller values for the last index indicate relatively high sample species richness. Beta diversity was used to compare gut microbiota compositions among groups. This analysis was performed by the unweighted pair group method and the arithmetic mean (UPGMA) clustering method based on weighted and unweighted UniFrac distances.

### Morphometry

Ileal tissue was fixed with 4% (v/v) paraformaldehyde, embedded in paraffin, and sliced into 5-μm sections. After deparaffinization, the sections were stained with hematoxylin and eosin (H&E) and observed under a light microscope. Ileal ultrastructure was captured by transmission electron microscopy (TEM).

### Immunohistochemical Analysis

Immunohistochemistry (IHC) was performed using antibodies against Toll-like receptor 4 (TLR4), nuclear factor κB (NF-кB), and tumor necrosis factor α (TNF-α). Zonula occludens-1 (ZO-1) and occludin (Abcam, Cambridge, UK) were labeled to delineate intestinal epithelial cell connections. Parafﬁn blocks were sliced (thickness: 5 μm), deparaffinized, and subjected to heat-induced antigen retrieval for 10 min. The sections were blocked with 3% (v/v) H_2_O_2_ for 15 min, incubated with primary antibodies at 4°C overnight, incubated with horseradish peroxidase (HRP)-conjugated secondary antibodies for 30 min at 37°C, stained with a diaminobenzidine (DAB) kit, counterstained with hematoxylin, and viewed under a light microscope at ×400. Observers were blinded to the treatments.

### Immunofluorescence

Paraformaldehyde-fixed ileum samples were embedded in paraffin blocks. Immunofluorescence double staining was used to label the M1 and M2 macrophages. Representative sections (thickness: 5 μm) were incubated overnight with the primary antibodies anti-inducible nitric oxide synthase (iNOS, Abcam, Cambridge, UK), anti-CD68 (Abcam, Cambridge, UK), and anti-arginase-1 (ARG-1, Cell Signaling Technology). CD68 is a surface macrophage marker. The iNOS and Arg-1 are markers of the M1 and M2 phenotypes, respectively. The samples were incubated with fluorescein-labeled secondary antibodies at 37°C and ﬂuorescence images were captured under a ﬂuorescence microscope (×400). At least three random sections were scored per ileum sample.

### Statistical Analysis

The data were processed in SPSS v. 19.0 for Windows (IBM Corp., Armonk, NY, USA). One-way ANOVA was used for statistical comparison of >3 treatment means. Tukey's *post hoc* test was run to contrast pairs of groups. Statistical significance was set at *p* < 0.05.

## Results

### SAL Prevented the Onset of T2D in ZDF Rats

**Figure 1:**
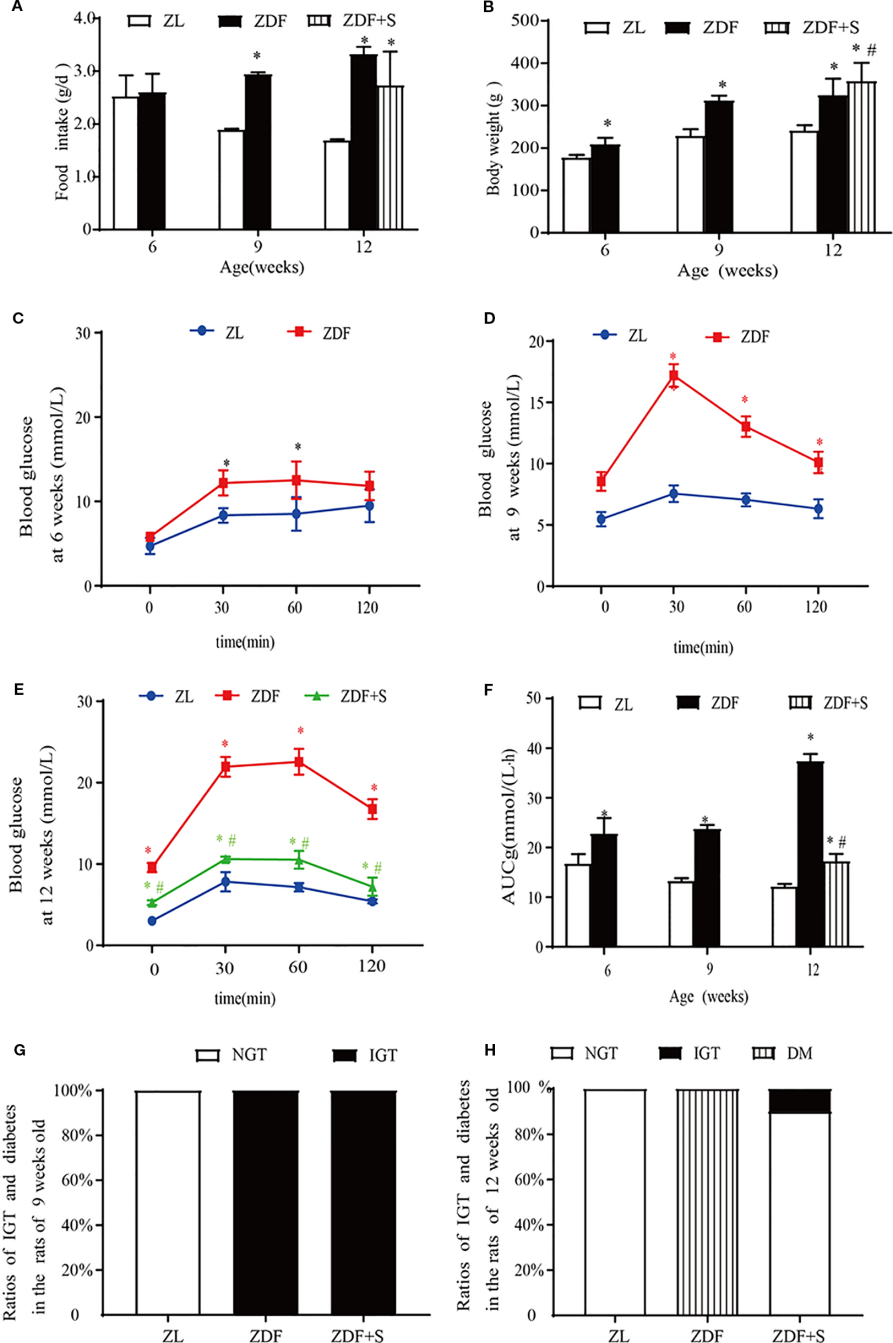
SAL treatment prevented the onset of diabetes in ZDF rats. **(A)** Serial changes in food intake. **(B)** Serial changes in body weight. **(C–E)** OGTT for rats age 6 weeks **(C)**, 9 weeks **(D)**, and 12 weeks **(E)**. **(F)** AUCg determined by OGTT. One-way ANOVA followed by Tukey's test compared differences between group pairs. Data are means ± SEM. *P < 0.05 vs. ZL rats; ^#^*P* < 0.05 vs. ZDF rats. **(G, H)** Ratios of IGT and diabetes in rats age 9 weeks **(G)** and 12 weeks **(H)**.

SAL administration without alteration in food intake (*p* > 0.05) increased the mean relative body weight of untreated ZDF rats (*p* > 0.05) (**Figures 1A, B**). ZDF rats had normal glucose tolerance at age 6 weeks but their blood glucose levels were comparatively higher at each measurement point than those of ZL rats (*p* > 0.05) (**Figure 1C**). The blood glucose levels of the ZDF rats continued to rise and developed into IGT by age 9 weeks (**Figure 1D**). At age 12 weeks, the blood glucose levels of the untreated ZDF rats were significantly higher at baseline and within 120 min than those of the ZL rats (*p* > 0.05). However, the corresponding blood glucose levels were significantly lower in the ZDF+S group (*p* > 0.05) (**Figure 1E**). The AUCg value illustrated this SAL-related hypoglycemic effect (*p* > 0.05) (**Figure 1F**). We calculated the ratios of rats with abnormal glucose tolerance to those with normal glucose tolerance based on OGTT results. All ZDF rats (100%) presented with IGT at age 9 weeks whereas all ZL rats had normal glucose tolerance (**Figure 1G**). All untreated ZDF rats developed T2D at age 12 weeks but only 10% of the SAL-treated ZDF rats progressed to IGT and none of them advanced to overt T2D (**Figure 1H**).

### SAL Improved Islet-Cell Function and Insulin Resistance in ZDF Rats

**Figure 2:**
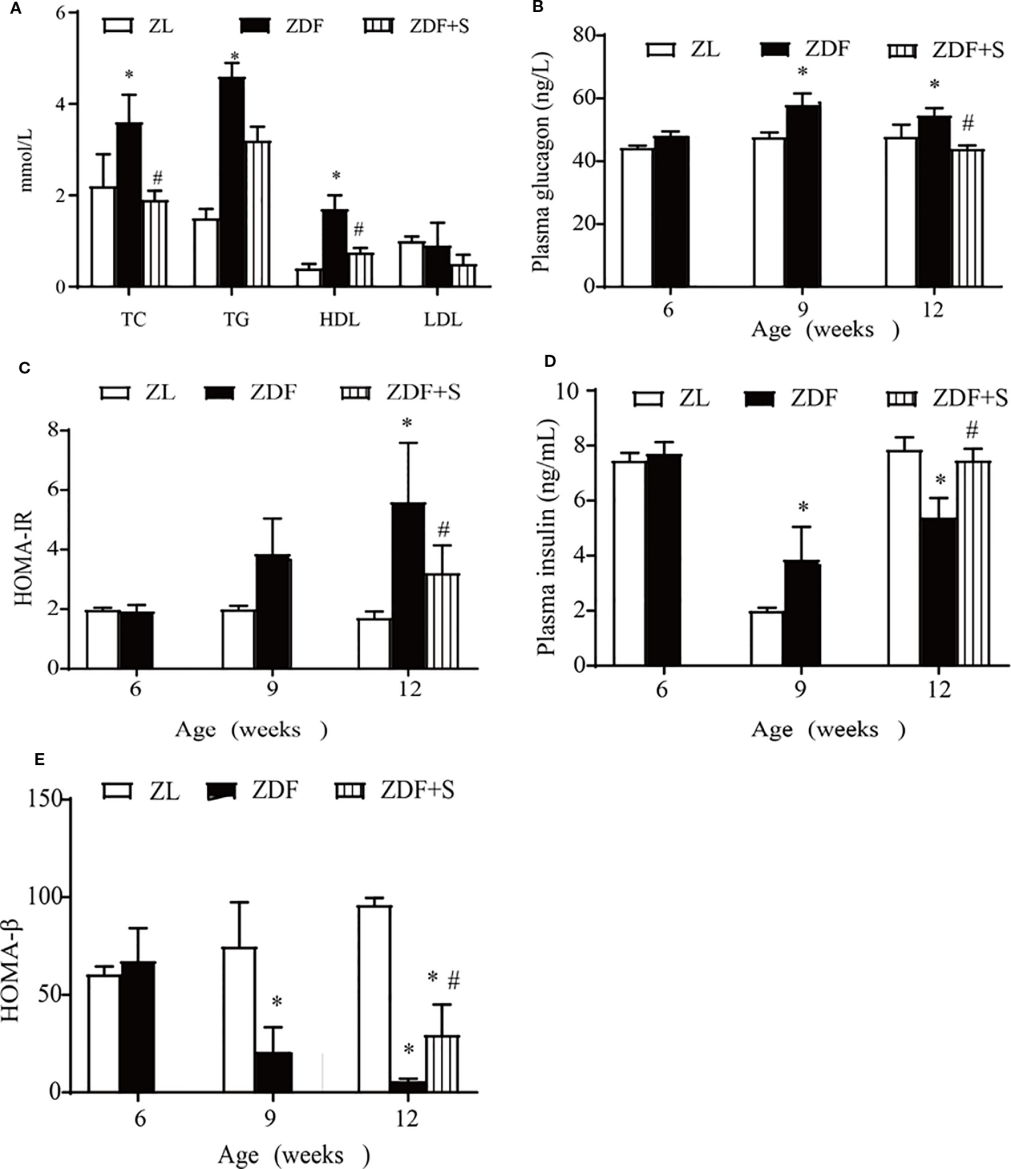
SAL improved islet-cell function and insulin resistance in ZDF rats. **(A)** Effects of SAL on lipid metabolism in rats age 12 weeks. Fasting plasma glucagon **(B)** and **(C)** HOMA-IR values in rats. **(D, E)** Insulin concentrations **(D)** and HOMA-β values **(E)** in rats. One-way ANOVA followed by Tukey's test compared differences between group pairs. Data are means ± SEM. *P < 0.05 vs. ZL rats; ^#^*P* < 0.05 vs. ZDF rats.

We examined the effects of SAL intervention on blood lipids and islet cell function. Compared with the untreated ZDF rats, those that underwent SAL intervention presented with reduced TC and HDL levels (*p* > 0.05) but no evident change in TG or LDL level (*p* > 0.05) (**Figure 2A**). Fasting plasma glucagon concentrations were increased in untreated ZDF rats at age 12 weeks but were significantly lowered in those receiving SAL (*p* > 0.05) (**Figure 2B**). Substantial insulin resistance was observed in the untreated ZDF rats but was significantly reduced by SAL according to the HOMA-IR values (*p* > 0.05) (**Figure 2C**). Fasting plasma insulin concentration was significantly increased in the ZDF rats at age 9 weeks but had markedly declined by 12 weeks. SAL intervention alleviated the low plasma insulin levels (*p* > 0.05) (**Figure 2D**). The HOMA-β values indicate β-cell function and were significantly reduced in untreated ZDF rats at 12 weeks (*p* > 0.05) but effectively maintained in the ZDF+S group (**Figure 2E**).

### SAL Treatment Restored Gut Architecture and Intestinal Epithelial Connections in ZDF Rats

**Figure 3:**
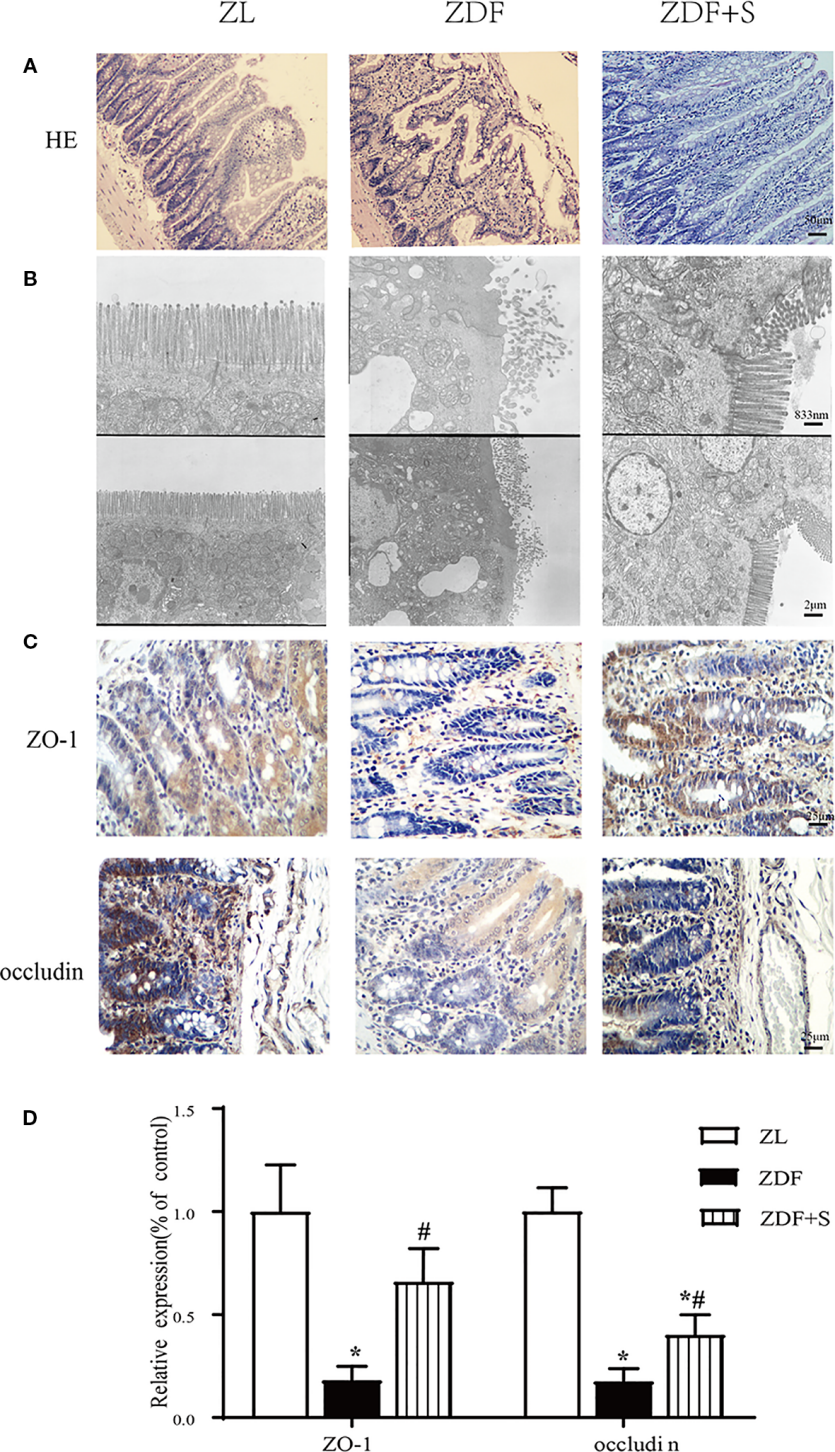
SAL treatment restored gut architecture and intestinal epithelial cell connections in ZDF rats. **(A)** H&E staining of rat ileum (original magnification: ×200; scale bar: 50 μm). **(B)** EM details of ileum structure in various groups (original magnification: ×5000 or ×12,000; scale bar: 2 μm or 833 nm). **(C, D)** Immunohistochemical quantification and analysis of ZO-1 and occludin expression (original magnification: ×400; scale bar: 25 μm). Error bars represent SEM. *P < 0.05 vs. ZL rats; ^#^P < 0.05 vs. ZDF rats.

Compared with the ZL rats, the ileum microvilli of the untreated ZDF rats were in disarray and significantly thicker and shorter. In contrast, the ZDF+S rats presented with elongated microvilli and essentially intact intestinal morphology (**Figure 3A**). The microvilli in the untreated ZDF rats were irregular, whereas those of the ZL rats were regular and had bundles of thin, striated filaments connected to the terminal web. In the untreated ZDF rats, the apices of the ileal enterocyte cytoplasms contained markedly swollen mitochondria with short cristae. The SAL treatment resulted in relatively less irregular microvillar membranes and mildly edematous mitochondria (**Figure 3B**).

The tight junction proteins ZO-1 and occludin are indicators of intestinal leakage. IHC staining showed that ZO-1 and occludin were diminished in the untreated ZDF rats compared to the other two groups at 12 weeks (*p* < 0.05). Moreover, the relative expression levels of ZO-1 and occludin were higher in the SAL intervention group than the untreated ZDF rats (*p* < 0.05) (**Figures 3C, D**).

### SAL Treatment Attenuates Intestinal Mucosal Inflammation in ZDF Rats

**Figure 4:**
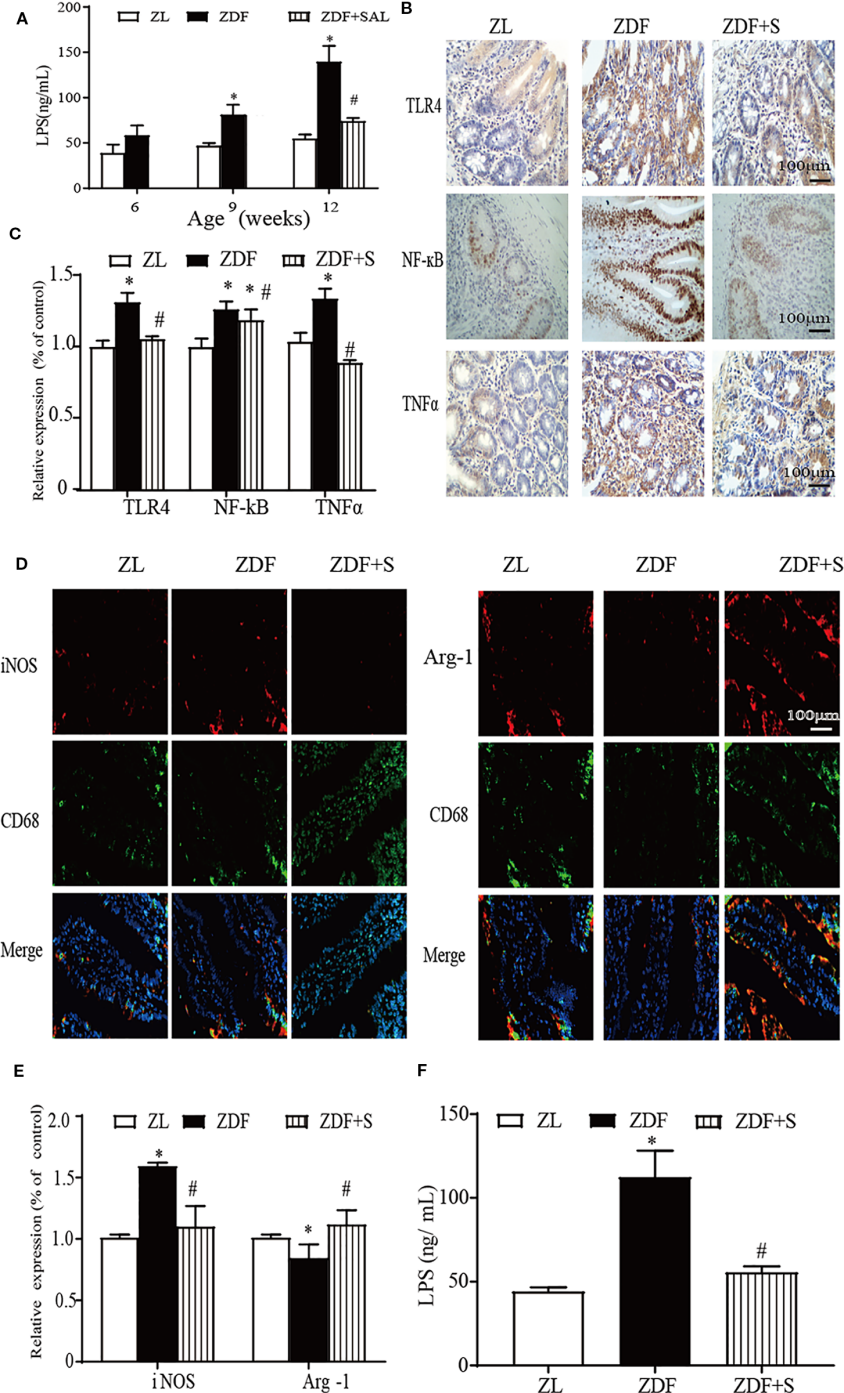
SAL treatment attenuated intestinal mucosal inflammation in ZDF rats. **(A)** LPS level in ileum extract supernatant. **(B, C)** Immunohistochemical (IHC) quantification and analysis of TLR4, NF-кB, and TNF-α expression (original magnification: ×400; scale bar: 100 μm). **(D, E)** Immunoﬂuorescence quantification and analysis of iNOS (represents M1 macrophage), Arg-1 (represents M2 macrophage) (original magnification: ×400; scale bar: 100 μm). **(F)** Serum LPS level. One-way ANOVA followed by Tukey's test compared differences between group pairs. Data are means ± SEM. *P < 0.05 vs. ZL rats; ^#^*P* < 0.05 vs. ZDF rats.

LPS are also known collectively as endotoxin. LPS are major constituents of the outer membrane of intestinal Gram-negative bacteria. Endotoxin is recognized as an important inflammatory stimulator (Chu et al., 2019). Here, the LPS level was elevated in the ileal extract supernatant of untreated ZDF rats but was comparatively lower for those treated with SAL (*p* < 0.05) (**Figure 4A**). TLR4, NF-кB, and TNF-α are inflammation biomarkers. As shown in **Figures 4B, C**, relative to the ZL rats, TLR4, NF-кB, and TNF-α were all upregulated in the untreated ZDF rats but downregulated after SAL intervention (*p* < 0.05).

LPS induce macrophages to polarize into various phenotypes. The two main macrophage phenotypes are M1 and M2 (Islam et al., 2018). M1 macrophages promote inflammation while M2 macrophages inhibit it. In untreated ZDF rats aged 12 weeks, the M1 macrophages were highly expressed. In ZL rats of the same age and the ZDF rats administered SAL, the M1 expression levels were relatively lower. The M2 macrophages were more highly expressed in the ZL and SAL intervention groups than the untreated ZDF group (*p* < 0.05) (**Figures 4D, E**). We also measured serum LPS levels to assess circulatory inflammation. Compared to the untreated ZDF rats, the SAL-treated rats showed significantly lower serum LPS levels (*p* < 0.05) (**Figure 4F**).

### SAL Treatment Increased Microbiota Diversity and Ameliorated Gut Dysbiosis in ZDF Rats

**Figure 5:**
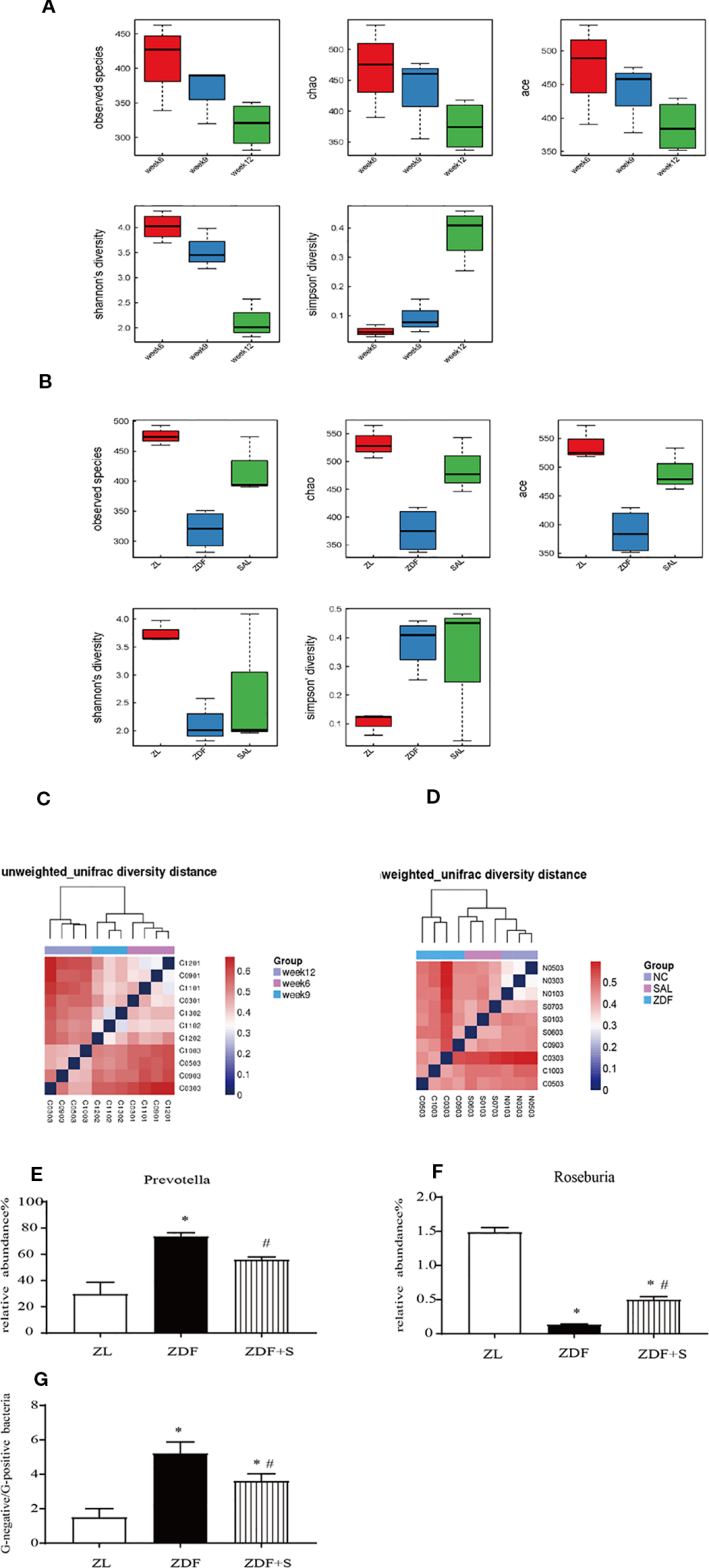
SAL treatment increased microbiota diversity and ameliorated gut dysbiosis in ZDF rats. **(A)** Analysis of microbiota α-diversity in feces of untreated ZDF rats at ages 6 weeks, 9 weeks, and 12 weeks. Data are means ± standard error of the mean (*n* = 3). **(B)** Analysis of microbiota α-diversity in feces of rats of various groups at age 12 weeks. Data are means ± standard error of the mean (*n* = 3). **(C)** Microbiota β-diversity in feces of untreated ZDF rats at ages 6 weeks, 9 weeks, and 12 weeks. **(D)** Microbiota β-diversity in feces of rats of various groups at age 12 weeks. **(E, F)** Relative genus-level gut flora abundance. **(E)** *Prevotella*. **(F)** *Roseburia*. One-way ANOVA followed by Tukey's test compared differences between group pairs. **(G)** Ratio of Gram-negative to Gram-positive bacteria in rats age 12 weeks. Data are means ± SEM. ^*^P < 0.05 vs. ZL rats; ^#^P < 0.05 vs. ZDF rats.

**Figures 5A, B** show that gut microbiota diversity declined with T2D progression but was restored by SAL administration. A β-diversity analysis showed that the relative differences in bacterial diversity between the NGT and IGT phases were small. Nevertheless, this difference had significantly increased by the DM phase (**Figure 5C**). In the 12-week untreated ZDF rats, the difference in bacterial diversity was significantly greater than it was for the ZL and ZDF+S groups. In contrast, the difference in bacterial diversity between the ZDF+S and ZL groups was not significant (**Figure 5D**).

The relative genus-level abundance of gut flora was evaluated for each treatment group. Compared to the ZL rats, the 12-week untreated ZDF rats presented with a greater and increasing abundance of *Prevotella*. SAL intervention reduced *Prevotella* levels compared with those observed for the untreated ZDF rats. Conversely, *Roseburia* abundance had decreased in the 12-week untreated ZDF rats but was restored by the SAL treatment (*p* < 0.05) (**Figures 5E, F**). We calculated the ratio of Gram-negative to Gram-positive bacteria in each rat group based on the relative abundances at age 12 weeks. The SAL intervention significantly corrected the imbalance in the ratio of Gram-negative to Gram-positive bacteria (*p* < 0.05) (**Figure 5G**).

## Discussion

Effective strategies must be formulated to lower the risk of developing T2D at the IGT stage. The present study was designed to assess the antidiabetic effects of SAL in ZDF rats and examine the related mechanisms. A recent study showed that intestinal flora disorders contribute to the development of T2D by inducing inflammation (Chavez-Talavera et al., 2017). SAL attenuates inflammation. Its recommended dose as an anti-inflammatory drug is 0.6–2.4 g day^−1^ (Knudsen and Pedersen, 2015). Other authors reported that SAL improves insulin resistance (Han et al., 2019a; Wang et al., 2019). Therefore, we speculate that low doses of SAL may mitigate inflammation by restoring order to the intestinal flora, thereby helping to prevent T2D.

Food intake was controlled in the ZDF+S group but the mean body weight was higher than that of the untreated ZDF group. This finding was consistent with that of a previous study (Chadha and Morris, 2015). SAL improved glucose metabolism in rats. All untreated ZDF rats developed T2D by 12 weeks whereas T2D was absent in their SAL-treated counterparts. Only 10% presented with IGT. Thus, SAL may prevent T2D in ZDF rats. Decreased β-cell function is typical of T2D and is accompanied by inflammation, changes in lipid metabolism, etc. The serum insulin levels of T2D model ZDF rats were high between ages 7 and 10 weeks but subsequently declined in response to β-cell failure (Chen and Wang, 2005). Our findings showed that SAL improved β-cell function in ZDF rats and corroborated the data of a previous report (Ariel et al., 2015). Our earlier research indicated that the underlying mechanism may include the prevention of β-cell dedifferentiation and the restoration of islet morphology/architecture (Han et al., 2019a).

Gut dysbiosis plays a vital role in the etiology and development of obesity (Ridaura et al., 2013), insulin resistance, and T2D (Qin et al., 2012). Changes in gut microbiota and specific microbial components influence metabolic endotoxemia and inflammation and alter intestinal permeability (Cani et al., 2007). Moreover, modifying gut microbiota and their components reduced intestinal permeability and strongly contributed to glucose homeostasis (Cani et al., 2007; Duparc et al., 2017). Cotillard et al. showed that populations with low microbial genome abundance (40%) were relatively more likely to present with pronounced dysmetabolism and low-grade inflammation (Cotillard et al., 2013). Improvement of gut microbiota with probiotics such as *Lactobacillus* genus (LAB) and *Bifidobacteria* helps prevent and treat obesity and T2D (Hampe and Roth, 2017). Probiotics may help prevent pathogen colonization by physically interfering with attachment, competing for nutrients, producing antimicrobial substances, and inactivating endotoxins (Medellin-Pena and Griffiths, 2009). Here, we found that SAL treatment increased the α- and β-diversity of fecal bacteria in HFD-fed ZDF rats. Therefore, the overall gut flora structure was improved by SAL intervention. Based on current and prior results, we conclude that SAL may help prevent T2D by improving intestinal flora.

We analyzed the relative genus-level abundance of gut flora. *Prevotella* is a potential proinflammatory bacterium that is closely associated with low-grade inflammation and insulin resistance (Pedersen et al., 2016; Larsen, 2017). Here, we found that SAL administration markedly decreased the relative abundance of *Prevotella*. *Roseburia* is a Gram-positive bacterium that produces short-chain fatty acids (SCFA) such as butyrate. It affects gut motility and helps maintain gut immune function (Tamanai-Shacoori et al., 2017). In the present study, the ratio of *Roseburia* was comparatively lower in T2D rats but was restored by SAL intervention. An earlier study reported that in obese and T2D patients, the ratio of Gram-negative to Gram-positive bacteria was relatively elevated and contributed to increases in serum LPS levels and low-grade inflammation (Qin et al., 2012). Compared with ZL rats, the untreated ZDF rats showed larger proportions of Gram-negative bacteria and fewer Gram-positive bacteria in their intestines. SAL intervention reduced the ratio of Gram-negative to Gram-positive bacteria. Thus, we proposed that the anti-inflammatory effect of SAL in ZDF rats was associated with alterations in gut microbiota structure.

T2D is a consequence of low-grade inflammation (Eguchi and Nagai, 2017). Increased proinflammatory cytokine synthesis and secretion promote inflammation (Gomes et al., 2017). Augmented LPS production in response to gut microbiota overgrowth may trigger proinflammatory responses (Xu et al., 2016). Bacterial death and decomposition impair intestinal barrier function and LPS is released into circulation. Thence, LPS triggers an inflammatory cascade, which, in turn, may lead to the development of early-onset inflammation and T2D (Xu et al., 2016). Impaired epithelial barriers permit LPS and endotoxins to leak into the bloodstream (“leaky gut syndrome”) (Han et al., 2019b). “Leaky gut syndrome” plays a crucial role in the pathogenesis of various diseases such as T2D (Xiao et al., 2014). Gut microbes may also modulate the expression of tight junction proteins such as ZO-1 and occludin and attenuate intestinal barrier function even further (Tran et al., 2015). ZO-1 and occludin protein expression and intestinal barrier function are impaired in diabetic humans and other mammals (Horton et al., 2014; Xiao et al., 2014). Here, rats administered a high-fat diet presented with ZO-1 and occludin downregulation. In contrast, ZO-1 and occludin were upregulated in the SAL intervention group and these responses may have partially contributed to the relatively lower insulin resistance observed in these rats.

**Figure 6:**
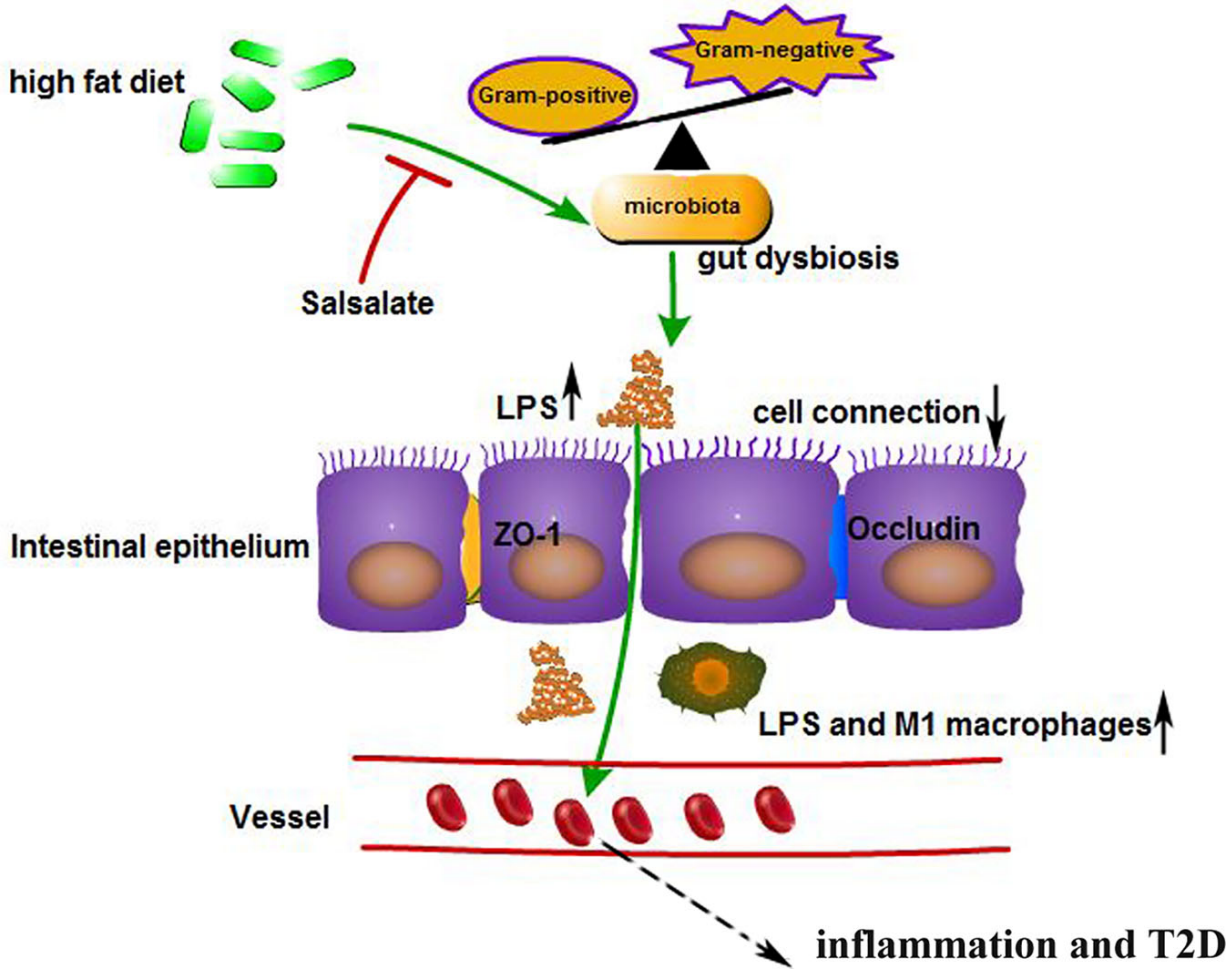
SAL treatment prevented onset of diabetes by suppressing inflammation and modulating gut microbiota. Intestinal flora status in response to high-fat diet and SAL intervention.

LPS is a component of the Gram-negative bacteria cytoderm. It is significantly correlated with the onset of T2D and low-grade inflammation (Wang et al., 2019). TLR4 is the main recognition receptor in human monocytes and is abnormally regulated during inflammation (Rakoff-Nahoum and Medzhitov, 2009). Upon activation, TLR4 augments macrophage activity *via* proinflammatory cytokines including TNF-α, IL-6, IL-1β, and NF-κB. In the present study, the 12-week untreated ZDF rats presented with notable increases in LPS concentration whereas SAL administration significantly decreased LPS in the ileum and serum. TLR4, NF-κB, and TNF-α were all relatively downregulated by SAL treatment. LPS may activate M1 macrophages and increase iNOS synthesis and secretion involved in inflammation-associated disorders (Rath et al., 2014). Conversely, M2 macrophages were reported to produce anti-inflammatory cytokines (Rath et al., 2014). SAL blocked excessive M1 macrophage production and restored M2 macrophage expression. The potential antidiabetic effects of SAL are mediated by its abilities to reduce intestinal inflammation and regulate macrophage phenotype. Based on the mentioned findings, we proposed a hypothetical mechanism by which SAL intervention may help prevent the onset of T2D (**Figure 6**).

There were still limitations in our study. Firstly, HOMA-β and HOMA-IR have been used to evaluate beta-cell function, as well as insulin resistance only in steady-state conditions. Advanced model-based techniques and other indexes of glucose tolerance will be used in our future study (Di Nardo et al., 2015; Morettini et al., 2017). This study demonstrated that SAL administration may prevent the onset of T2D in ZDF rats by modulating overall gut microbiota composition, increasing the proportion of probiotics, maintaining intestinal integrity and permeability, and reducing intestinal inflammation. Here, we elucidated the effects of intestinal microflora disorder on the pathogenesis of T2D and the role of SAL in preventing this condition.

## Data Availability Statement

The sequencing data for this study can be found in the GenBank with the accession no. MN474033-MN475147.

## Ethics Statement

This study was carried out in accordance with the Animal Use Guidelines of the Tianjin Medical University Committee. The protocol was approved by the Animal Use Committee of Tianjin Medical University.

## Author Contributions

CS, JY, and BC conceived the project. XZ and XC performed the experiments and wrote the entire manuscript. XJ analyzed results and interpreted the data. FH and JW revised the manuscript. XY, JX, ZG, XL, and MZ participated in part of the work. All authors read and approved the final manuscript.

## Funding

This work was supported by the National Key R&D Program of China [2018YFC1314000], the National Natural Science Foundation of China [81603461 and 81774043], the Natural Science Foundation of Tianjin City [17JCZDJC34700 and 17ZXMFSY00140], the Tianjin Health Bureau Foundation [16KG167], the Scientific Research Funding of Tianjin Medical University Chu Hsien-I Memorial Hospital [2015DX05 and 2018RC02], and Fund of the State Key Laboratory of Kidney Diseases in PLA General Hospital (KF-01-133).

## Conflict of Interest

The authors declare that the research was conducted in the absence of any commercial or financial relationships that could be construed as a potential conflict of interest.
